# Hydrophobia

**Published:** 1889-06-01

**Authors:** 


### HYDROPHOBIA.

HYDROPHOBIA, the literal meaning of which is fear of water (this being one of the prominent symptoms of the malady), is unfortunately sufficiently common in European countries to have recently attracted a considerable amount of both professional and public attention. It is, however, we think, more frequently seen in the Eastern hemisphere, and especially in India. The line from Horace, "Hac rabiosa fugit canis, hac lutulenta ruit sus," is applicable to most villages and many towns in India, where semi-wild pariah dogs swarm, which the Hindus, from religious motives, will not kill. It is true there are no reliable statistics proving the greater prevalence of hydrophobia in the East, and it has been stated that heat of climate does not conduce to rabies in the dog, notwithstanding that we are accustomed to speak of the "dog-days." It is certain that dogs do become rabid in the coldest weather. It is also almost as certain that rabies is only communicated directly from one animal to another. Personally, however, we think atmospheric heat does predispose to rabies. But this question is of minor importance, for the prevalence of hydrophobia in India may be otherwise accounted for. It has been already stated that Indian villages abound with semi-wild pariah dogs, which the natives will not destroy. And in addition to this ever-present source of danger, hydrophobia may not only arise from the bites of dogs, cats, and foxes, but also of jackals, wolves, mongooses, and even camels, animals not ordinarily found in European countries, all of which are subject to rabies. Several instances of mad jackals attacking and injuring groups of people are on record. There is reason to believe that many cases of hydrophobia occur among the vast population of the Eastern Empire which are either not recorded at all, or are wrongly recorded. For although the extension of hospitals and dispensaries throughout the land has been marvellously great and rapid, there are still extensive tracts and thousands of villages which have not yet come under the influence of the ever-increasing system of medical aid. The question, however, of the country in which hydrophobia is most prevalent is again a minor one, seeing that the malady is sufficiently common in most lands to have attracted the attention noted above.

After hearing so much of late about hydrophobia and its treatment, the public would doubtless be surprised to learn that the very existence of such a disease has been questioned from very early times. Cœlius Aurelianus, A.D. 30, raised the query whether hydrophobia is an affection of the body or mind. Years back Sir Isaac Vennington, Regius Professor of Physic at Cambridge, denied that hydrophobia existed at all. Bosquillon, a professor of medicine, and physician to the Hotel Dieu, was utterly sceptical as to the existence of any virus. Professor Duck, of Edinburgh, held that hydrophobia in man is not the result of any poison. Demangeon observed, if the hydrophobia virus has any existence, which seems highly problematical, it must be admitted that its effects do not differ in a single characteristic sign from those of the imagination. In 1827, Garard asserted there was no specific hydrophobia poison. Still more recently Dr. Dulles gave his view that it is not a specific disease arising from the bite of a mad dog. Several other writers might also be quoted to the same effect. The non-existence of hydrophobia cannot however be accepted. According to Hecker hydrophobia was known at least 400 years B.C. It is also alluded to in the works of Aristotle, Xenophon, Plutarch, Virgil, Horace, Ovid, Celsus and Galen. In Holland's Plutarch there is the following passage: "Athenodorus in the first book of his epidemiale, or popular diseases, writeth, that not only the *leprosie*, but also the *hydrophobie*, occasioned by the biting of a mad dog, were first diagnosed in the days of Asclepiades." In modern times the symptoms of the disease have been so fully investigated that no doubt of the existence of the malady remains in the minds of the scientists of the medical profession. Moreover, the occasional occurrence of the malady in children, even in infants and in lunatics, should be conclusive. If not, there is the fact that it has been artificially excited in animals by inoculation with hydrophobic virus.

The reason why the existence of a specific disease has been denied is doubtless to be found in the fact that there is such a malady as *pseudo*, or false hydrophobia, or, as it has been termed, "lyssophobia." The authors mentioned above, and others, denying the existence of hydrophobia, attribute the symptoms arising after the bite of a dog to mental emotion or fear. Dr. Dolan, in his work on hydrophobia, admits that mental excitement may terminate in a spurious hydrophobia, presenting symptoms somewhat resembling the genuine disease. This he regards as a distinct nervous affection, or, as Trousseau termed it, "mental" hydrophobia. Roberts, in his "Practice of Medicine," mentions that a patient who has been bitten by a supposed mad dog may imagine all kinds of symptoms simulating hydrophobia. Cases are on record where the disease followed the bite of a dog which did not at the time, nor for several weeks afterwards, present symptoms of rabies. But to account for such a case, it is possible that rabies may affect a dog as a latent and not recognisable malady, yet the dog may be able to convey it. Still more extraordinary are the facts that symptoms of the disease have appeared in, and even death has occurred to, persons bitten by a dog, which remained perfectly well afterwards, and to persons who have never been known to have been bitten by a dog or any other animal. Years back the celebrated and accurate Pinel gave the history of such a case, and there is another recorded by Savirotte in the *Journal des Savans* for August, 1757. Cases of spurious hydrophobia are mentioned in the *Lancet*, July 17, 1886; in the *Trans. Clin. Soc.*, 1883; in the *Lancet*, January, 1883; a case of "phantom hydrophobia" in the *Medical Times*, 1882, p. 46, and in various other places. Chevers cites two cases of persons bitten by dogs, who, constantly declaring they would go mad, at last did so.

There is no doubt whatever that after a dog bite, whether the animal is mad or not, deaths take place from fear—just as occasionally results after snakebite, or from the fear of a surgical operation, or even from joy. The influence of fear or mental excitement in checking the secretion of bile and causing sudden jaundice, in altering the secretion of milk and rendering it injurious to the sucking child, in determining an attack of cholera, are facts well known. Dr. Tuke, in his recently-published "Influence of Mind over Body," when writing on the heart, mentions that Hunter, who was subject to "spasms in his vital parts," said, "My life is at the mercy of any scoundrel who chooses to put me in a passion." Cases have been recorded when the sight of wounds has caused sensation, swelling, and even abscess in the same position in the person of the individual witnessing it. Sir J. Paget was consulted in four instances within the fortnight following the death of Napoleon III. by persons who experienced sensations of stone in the bladder, when there was no stone. Mental emotion has also been known to have been followed by different forms of paralysis, and by convulsions, as "revivals" testify. An instance has been recorded of a person being given air to inspire instead of chloroform, falling into a sleep, during which several teeth were painlessly extracted. The person having had chloroform before, went to sleep under suggestion, as explained by Dr. Lloyd McKey in his late work, "Psycho-Therapeutics; or, Treatment by Sleep and Suggestion." As nearly everyone knows more of less of the symptoms of hydrophobia, the spurious form originating in fear or mental emotion simulates the symptoms of the real disease — just as after snakebite death has occurred from syncope, the result of fear. If the person is naturally nervous or hysterical, spurious hydrophobia will be more likely. Lewes, in "Problems of Life and Mind," remarks, "Thus, if I have been bitten by a dog when I pinched its tail, I infer that the next time I pinch a dog's tail he will try to bite me." This is a process of mental induction. When an individual is bitten by a mad dog, or even any dog, knowing that persons have suffered from hydrophobia after such bites, the person injured, unless very strong-minded, fears and infers the disease, which, if not really occurring, is simulated. The influence of emotion in causing spasm of the muscles of the glottis and larynx is well illustrated by a case given by Tuke; and Trousseau remarked that laryngismus stridulus generally comes on under the influence of mental emotion or fright. We have every day examples of hysteria simulating various maladies. The hypochondriac usually fancies himself the subject of various bodily ills, and does not believe if told there is little the matter with him. Hydrophobia is certainly not the only malady of which spurious symptoms are caused by the imagination, which means fear, anxiety, mental emotion, and constant dwelling of the mind on the subject. As Pope has it:

> "Men prove with child as powerful fancy works,
> And maids turned bottles, cry aloud for corks."

As further demonstrating the influence of mind and fear, a case given by Zeimssen may be mentioned. A boy was followed by a street rabble who charged him with being hydrophobic. The boy was so frightened that symptoms of hydrophobia appeared, when the youth was sent into the country and quickly recovered. On his return to town exactly the same circumstances recurred. When a wound has been inflicted by a really rabid animal, emotion may be the cause of the outbreak of hydrophobia. It is stated on the authority of "Memoirs of the Royal Society of Science, Montpellier," that two brothers were bitten by a mad dog, one of whom went to Holland. On returning ten years afterwards and learning his brother had died of hydrophobia, he was seized and also died. It should be recollected that extreme mental agitation and terror, also spectral delusions, are characteristics of true hydrophobia. And as showing the power of the mind, even in true hydrophobia, it may be remarked that cases in children, and in persons who have no suspicion of the disease, are of a milder character.

If diseases were always characterised by typical symptoms, there would not be much difficulty in diagnosing true hydrophobia from anything else. But it is comparatively seldom that all the signs and symptoms are fully manifest. Although most observers agree in the main phenomena of dread of liquids and of air, secretion of viscid mucus, and hawking and spitting with paroxysmal attacks of a spasmodic nature, still, the variety in minor points is often such that hydrophobia may, like many other diseases, be designated a Protean malady. Cases have occurred (*vide Madras Medical Journal*, No. 13) in which fear of, and spasm from, drinking water were not strongly marked, and ceased altogether in the later stages. The principal diagnostic marks between true and false hydrophobia would appear to be that the throat spasms of the latter malady are not of the true respiratory character, but are really *dysphagia*, without that peculiar catching of the breath marking hydrophobia. Such *dysphagia* may sometimes be overcome by an effort. It is also stated there is not the extreme irritability of surface in false hydrophobia which characterises the true disease. If the affection lasts more than four days the probability is that it is not true hydrophobia. A case is on record where the sufferer was told his malady had lasted too long, whereupon he quickly recovered. The diagnosis is, however, occasionally so uncertain that the absence or presence of marks of bite is allowed to decide the matter.

The question is complicated in another way. It cannot be denied that various maladies have been mistaken for hydrophobia. Inflammation of the œsophagus has given rise to symptoms somewhat resembling it. Also a phase of intermittent fever, from its presenting peculiar symptoms, has been termed "febris intermittens hydrophobiœ." Cerebo-spinal fever, again, sometimes presents symptoms very like hydrophobia. The similarity of tetanus is well known. Although the diagnosis of typical cases is sufficiently easy, tetanus in an advanced stage sometimes presents a perfect picture of hydrophobia. Thus there has been described a tetanic form of hydrophobia and a hydrophobic form of tetanus—so with poisoning by strychine there may be a similarity. Hysteria, among others of its vagaries, occasionally mimics the disease. We have in recollection a case in which the symptoms were sometimes typical of hydrophobia, at others typical of tetanus, although it could not be discovered that the sufferer had acquaintance with the phenomena of either affection. The history of the case, the age, sex, and various hysterical manifestations cannot fail in establishing a correct diagnosis in such a case. A combination of insolation and delirium tremens, so often seen in the East, is frequently further complicated by symptoms resembling those of hydrophobia. In epilepsy, as well as hysteria, peculiar affections of the organs of deglutition are sometimes witnessed strikingly similar to hydrophobia. In the absence of history, hydrophobia may be mistaken for acute mania or *vice versa*, for in the insane there is sometimes developed a dread of water and even salivation, characterised by a reckless ejection of saliva. In short, it may be correctly asserted that hydrophobia, or at least its principal symptoms, occur as a manifestation of various diseases.

The fear of hydrophobia after dogbite is so general that the majority of the public would doubtless feel surprised to learn that although there is sufficient evidence to show that a very slight scratch or wound inflicted by a rabid animal may be followed by hydrophobia, it is not a necessary sequence that every person injured by a rabid animal must develope the malady.

(*To be continued.*)

